# Role of complement factor D in cardiovascular and metabolic diseases

**DOI:** 10.3389/fimmu.2024.1453030

**Published:** 2024-10-02

**Authors:** Yingjin Kong, Naixin Wang, Zhonghua Tong, Dongni Wang, Penghe Wang, Qiannan Yang, Xiangyu Yan, Weijun Song, Zexi Jin, Maomao Zhang

**Affiliations:** ^1^ Department of Cardiology, The Second Affiliated Hospital of Harbin Medical University, Harbin, China; ^2^ The Key Laboratory of Myocardial Ischemia, Harbin Medical University, Ministry of Education, Harbin, China

**Keywords:** CFD, adipsin, inflammation, cardiovascular disease, metabolic diseases

## Abstract

In the genesis and progression of cardiovascular and metabolic diseases (CVMDs), adipose tissue plays a pivotal and dual role. Complement factor D (CFD, also known as adipsin), which is mainly produced by adipocytes, is the rate-limiting enzyme of the alternative pathway. Abnormalities in CFD generation or function lead to aberrant immune responses and energy metabolism. A large number of studies have revealed that CFD is associated with CVMDs. Herein, we will review the current studies on the function and mechanism of CFD in CVMDs such as hypertension, coronary heart disease, ischemia/reperfusion injury, heart failure, arrhythmia, aortic aneurysm, obesity, insulin resistance, and diabetic cardiomyopathy.

## 1 Introduction

Cardiovascular and metabolic diseases (CVMDs) constitute the principal causes of death globally. Cardiovascular diseases (CVDs) constitute more than two-thirds of the deaths associated with a high body mass index (BMI) (1). The pathogenesis of these diseases remains incompletely clarified. Although researchers have made great progress in the prevention and treatment of CVMDs, the morbidity, disability and mortality rates remain high.

The development and prognosis of CVMDS are closely related to adipose tissue. For each 5 kg/m^2^ increase in BMI, the risks of CVDs will increase varied from 10% to 49% (2). Adipose tissue is an energy storage organ as well as an active and inflammatory organ capable of releasing adipokines (3). Adipokines modulate lipid metabolism, glucose metabolism, inflammation, and blood pressure. CFD is a serine protease that is produced mainly by adipose tissue and macrophages. CFD is an essential rate-limiting enzyme in complement alternative pathway (AP). As an adipokine, CFD regulates metabolism by increasing adipocyte differentiation and lipid accumulation, protecting beta-cells, and promoting insulin secretion. As a complement factor, CFD amplifies complement cascade activation and protects cells from infection, while it induces low-grade inflammation (LGI) in CVDs.

Mounting evidence suggests that CFD plays a significant role in CVMDS by regulating immune system homeostasis and metabolic balance. CFD levels are implicated in the etiology of several CVMDs. In a large population-based cohort, participants with higher CFD have a greater adverse cardiovascular profiles (4). CFD is positively associated with CVDs incidence. For each 1 standard deviation increase in the plasma concentration of CFD, the incidence of CVDs is 15% greater (5). Increasing CFD levels are correlated with obesity, hypertension, LGI, and endothelial dysfunction (6). However, CFD often acts as a protective factor in diabetes and diabetic cardiomyopathy.

**Figure 1:**
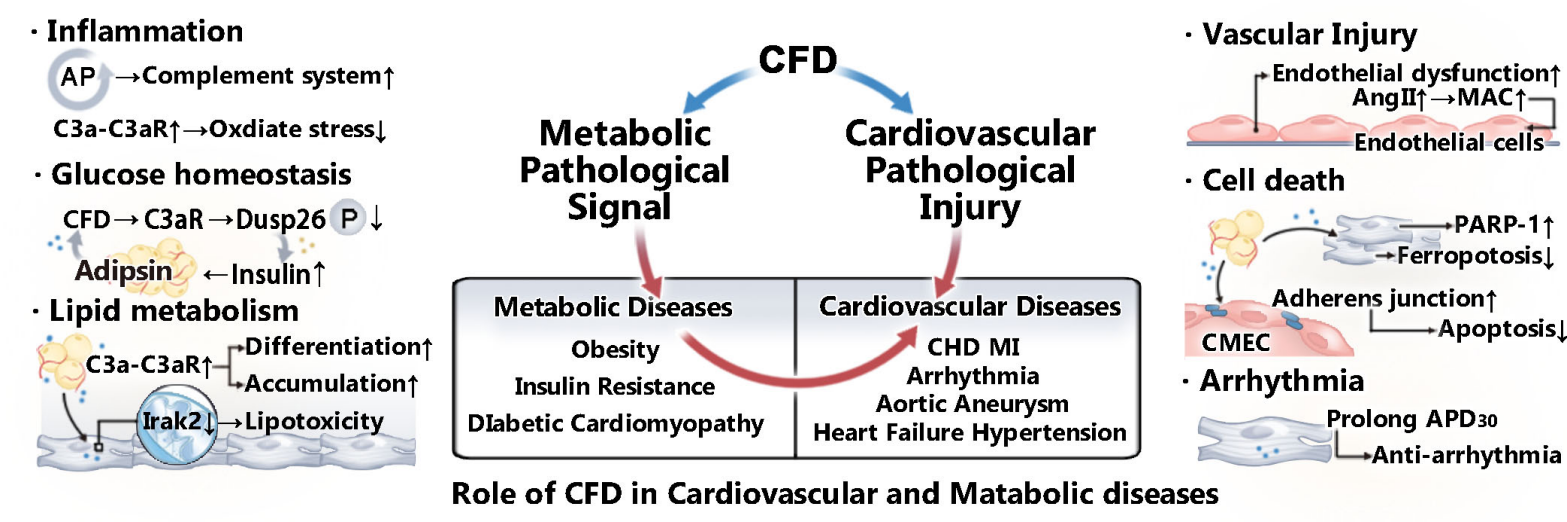
Role of CFD in CVMDs. CFD plays a role in various cardiovascular and metabolic diseases by regulating metabolic pathological signals and mediating cardiovascular pathological injury. CFD is closely related to the activation of the complement system. In addition, as an adipokine, it plays a vital role in lipid metabolism and glucose homeostasis. Moreover, studies have shown that CFD is associated with vascular injury, apoptosis, and arrhythmia.

Given the controversial role of CFD in different contexts of cardiovascular system immunity and metabolism (**Figure 1**), summarizing the specific effects of CFD on CVMDs is crucial for further research. In this review, we summarize the progress of CFD in CVMDs in recent years.

## 2 Introduction of CFD

### 2.1 The structure and maturation of CFD

CFD is a differentiation-dependent serine protease with a molecular weight of 24.4 kDa. Two variants of immature human CFD exist: variant 1, which is composed of 251 amino acids with an 18-amino acid leader sequence, and variant 2, which is composed of 260 amino acids with a 25-amino acid leader sequence (7). Apart from the pro-peptide, the two variants have the same polypeptide chain and form the same mature pro-CFD. As an inactive zymogen, pro-CFD consists of 235 amino acids and matures after the cleavage of a 6-amino acid peptide. Activated human CFD consists of a single serine protease domain of 228 amino acids with a catalytic triad (7). This process seems to occur during or immediately after secretion (8). Over 99% of plasma CFD exists in the form of CFD instead of the pro-CFD form (9). Human CFD is not glycosylated. In contrast, mouse CFD is highly glycosylated, possessing an molecular mass of approximately 42 and 45 kDa and being more stable in circulation (7, 10).

CFD circulates in a self-inhibited form and possesses relatively low proteolytic activity in blood (11). CFD has a narrow substrate specificity. CFD only binds to an open complement factor B (CFB) conformation in its Mg^2+^-dependent complex with C3b, triggering reversible conformational changes in the catalytic center of CFD (12). After the cleavage of CFB, CFD returns to an inactive resting-state conformation in circulation (13).

### 2.2 The production and metabolism of CFD

CFD is primarily and constitutively secreted by adipocytes, is also produced by macrophages and monocytes, and is synthesized by a small number of brain astrocytes (14). Among all the complement components, CFD has the lowest level in blood, ranging from 1 to 2 μg/mL, with no difference between healthy men and women (15). The CFD level in heart tissue depends on its serum level, whereas heart tissue expresses CFD mRNA at relatively low levels. Encircling the heart and blood vessels, epicardial adipose tissue (EAT) and pericardial adipose tissue (PVAT) synthesize CFD and deliver it to the heart via exosomes (16, 17). CFD is filtered through the glomerulus and then reabsorbed and decomposed by the renal tubules (7). It is estimated that the fractional catabolic rate of plasma CFD is 60% per hour, resulting in a lower serum concentration under nonpathological conditions (18). However, serum CFD levels can be influenced by renal dysfunction (19). CFD plasma levels increase approximately tenfold in patients with end-stage renal failure (20). However, CFD levels increase in the urine and decrease in the plasma of patients with Fanconi syndrome because of selective impairments in the renal tubular epithelium (21).

### 2.3 The physiological role of CFD

The role of CFD in protecting cells from infection has been well recognized. CFD deficiency causes insufficient terminal C5b-9 complement complex (TCC) generation, which ultimately increases the risk of meningococcal infection approximately 6000-fold (22). In families with CFD deficiency, family members show low AP activity and have meningitis and pneumonia (22, 23). CFD is also involved in other physiological processes. In previous studies, evidence indicating a central role of CFD in energy homeostasis and systemic metabolism has been put forward. CFD promotes the differentiation of adipocytes and the accumulation of lipids by activating the C3a-C3aR pathways in adipocytes and preadipocytes (24–26). In addition, CFD promotes the generation of acylation-stimulating protein (ASP) by catalyzing the conversion of C3 to C3a. As a C3 cleavage product of exopeptidase activity, ASP regulates lipid storage by increasing the activity of diacylglycerol O-acyltransferase 2 (27). CFD, which is upregulated by proliferator-activated receptor gamma (PPARγ) acetylation, inhibits Wnt/β-catenin signaling and thus primes bone marrow mesenchymal stem cell adipogenic differentiation in mice (28). As a diurnal factor, CFD plays an adipose-autonomous role in metabolic rhythms. In mouse adipose tissue, the expression of CFD exhibits a rhythm pattern, with a peak at zeitgeber time (zt) 12 and a trough at zt0 (29). No fluctuations in CFD are observed in the plasma (29). PPARγ acetylation upregulates CFD transcription, which destabilizes BMAL1 and mediates metabolic rhythms (29). CFD also plays a role in glucose metabolism. Several studies have indicated that CFD promotes insulin secretion and sustains β-cell function via the C3a-C3aR1 pathway (30).

### 2.4 The pathophysiological role of CFD

CFD contributes to paroxysmal nocturnal hemoglobinuria (PNH), atypical hemolytic uremic syndrome (aHUS), geographic atrophy (GA), and various diseases (**Table 1**). Patients afflicted with PNH and aHUS exhibit excessive activation of the AP. CFD inhibitors decrease C3 fragment deposition on PNH erythrocytes and abate complement-mediated hemolysis (31). Complement dysregulation is a key component of the pathogenesis of systemic lupus erythematosus (SLE). MBL-associated serine protease (MASP)3 is likely a direct activator of pro-CFD in resting human blood, cleaving inactive pro-CFD into active CFD (32). Compared with healthy individuals, SLE patients exhibit increased MASP-1 and MASP-3 levels. In addition, CFD, CFB and MASP1/3 are deposited along the glomerulus in patients with lupus nephritis (LN), which demonstrates that AP is involved in the pathogenesis of LN (33).

**Table 1:** Summary of the main studies investigating the effects of CFD in multiple diseases.

Disease	Species	Characteristic	Treatment	Finding	PMID
CKD	Human	a. 30 healthy subjects b. 30 CKD III & IV participants c. 30 CKD III & IV renal transplant recipients	NA	CFD levels are increased in the microparticles of CKD and transplant patients, which also activates the alternative pathway in serum.	30006493
PNH and aHUS	Human Monkey	a. 3 PNH patients & 4 aHUS participants b. 3 Monkeys	CFD inhibitor ACH-4471	ACH-4471 inhibits complement-mediated hemolysis in patients and blocks the AP activity in monkeys.	27810992
C3G	Human	29 C3G participants	NA	Patients CFD concentrations are inversely correlated with eGFR. AP are systematic activated.	36404708
TMA-associated LN	Human	a. 79 LN-associated TMA participants b. 79 LN participants without TMA	NA	TMA has stronger staining CFD deposited along the glomerulus than LN patients.	36582241
GA	Human	a. 42 GA participants b. 41 GA participants c. 40 GA participants	a. Lampalizumab injection monthly b. every other month injection c. sham control	Lampalizumab treatment reduce lesion area progression.	28637922
GC	Human	4 GC-derived cell lines	NA	All cell lines secret a large amount of CFD.	12095175
Breast cancer	Human Mouse	CFD-KO mice and mADSCs	NA	In mADSCs, CFD-KO reduces its ability to enhance the proliferation of PDX cells, and addition of CFD can restore this ability.	34439392
IA	Mouse	FF mice	NA	FF mice are resistant to IA and bone destruction because of a lack of CFD.	31167128
NAFLD	Human	908 NAFLD participants	NA	CFD can predict NAFLD remission independently.	34814152

In addition to cardiovascular and metabolic diseases, CFD is also involved in various diseases such as PNH, aHUS, GA, chronic kidney disease, and tumors. Its related pathogenic mechanisms and roles have been widely studied. CKD, chronic kidney disease; TMA, thrombotic microangiopathy; LN, lupus nephritis; GC, gastric cancer; KO, knockout; mADSCs, mammary adipose tissue-derived stem cells; PDX, patient-derived xenograft; IA, inflammatory arthritis; FF, fat-free; NAFLD, nonalcoholic fatty-liver disease.

CFD, together with MASP-1, C3, and C5, participates in the hemostatic response. While playing a protective role in preventing bleeding, it also exacerbates thrombotic and inflammatory diseases. While research has shown that platelets secrete CFD and activate AP (34), research has shown that CFD deficiency in mice can prevent thrombosis, and the inhibition of CFD can reduce platelet activation during vascular injury (35). A decrease in CFD in heparinized human whole blood can inhibit platelet activation in a simulated extracorporeal circulation circuit (36).

**Figure 2:**
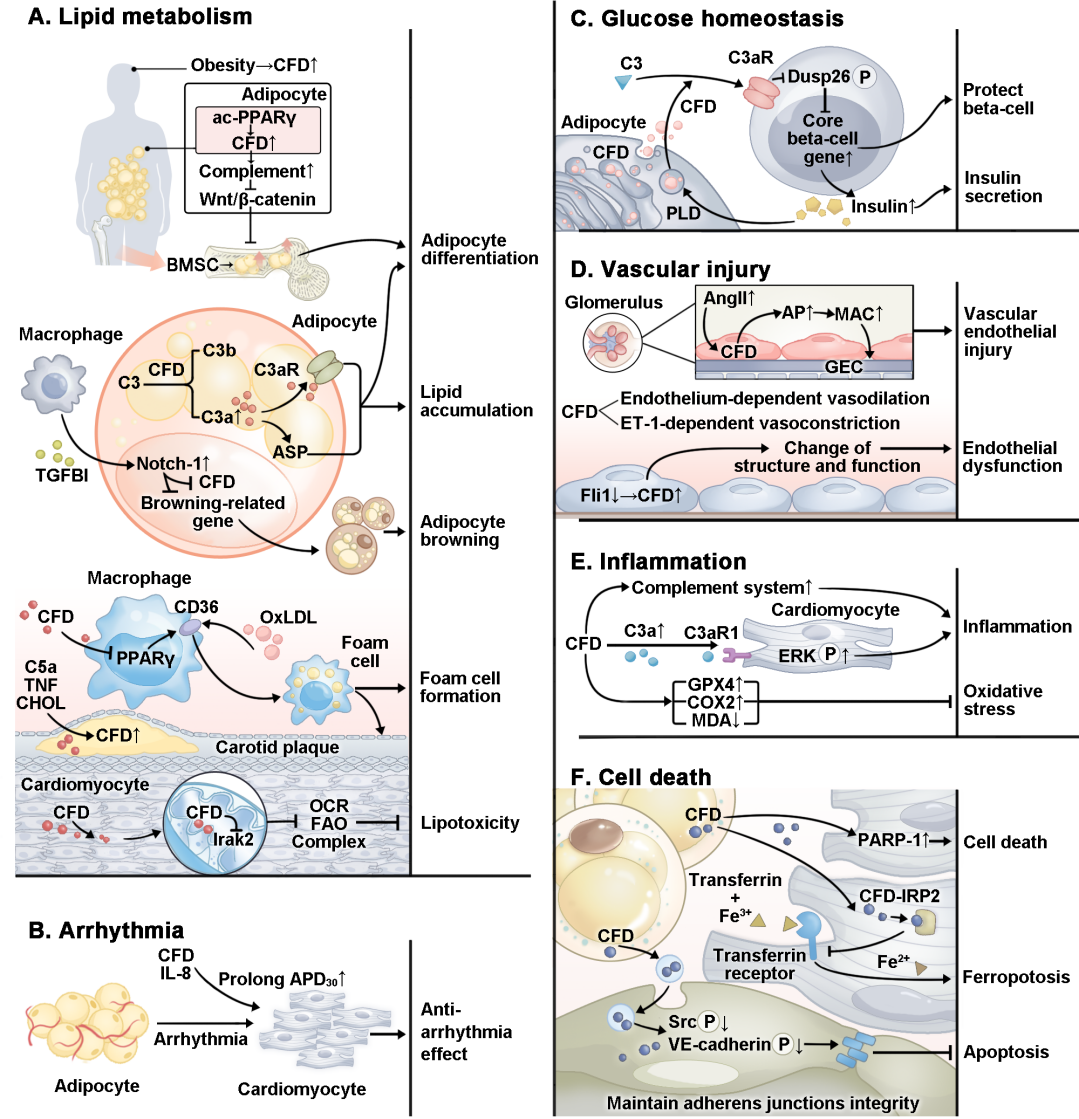
Specific mechanism by which CFD plays a role in CVMDs. In CVMDs, CFD plays a complex role. **(A)** CFD regulates adipocyte differentiation and lipid accumulation through multiple pathways. CFD promotes foam cell formation. However, in cardiomyocytes, CFD inhibits lipotoxicity. **(B)** CFD plays an antiarrhythmic role by prolonging APD_30_. **(C)** CFD protects beta-cells and promotes insulin secretion. **(D)** CFD is related to vascular endothelial injury and endothelial dysfunction. **(E)** CFD plays a pro-inflammatory role through the complement system and multiple pathways. In addition, studies have reported that CFD inhibits oxidative stress. **(F)** CFD protects cardiomyocyte against ferroptosis and maintains the integrity of cardiac microvascular endothelial cell adherens junctions. However, CFD activates PARP-1 and causes cardiomyocytes apoptosis. ac, acetylation; PPARγ, proliferator-activated receptor gamma; TGFBI, transforming growth factor-beta; OxLDL, oxidized low-density lipoprotein; TNF, tumor necrosis factor; CHOL, cholesterol; Irak2, interleukin-1 receptor-associated kinase 2; OCR, oxygen consumption rate; FAO, fatty acid oxidation; IL-8, interleukin 8; APD, action potential duration; Dusp26, dual specificity phosphatase 26; P, phosphorylation; PLD, phospholipase D; AngII, angiotensin II; GEC, glomerular endothelial cell; Fli1, friend leukemia virus integration 1; ERK, extracellular signal-regulated kinase; GPX4, glutathione peroxidase 4; COX2, cyclooxygenase-2; MDA, malondialdehyde; PARP-1, poly ADP-ribosepolymerase-1; IRP2, iron regulatory protein 2; Src, proto-oncogene tyrosine-protein kinase; VE, vascular endothelial.

CFD plays an important role in CVMDs through various pathological mechanisms (**Figure 2**), which we will discuss in detail later.

## 3 CFD and the complement system

### 3.1 General description of the complement system

Complement system activation involves three pathways: the classical pathway (CP), the lectin pathway (LP), and the AP (37). The activation of the CP is driven mainly by antigen-antibody complexes as well as C-reactive protein and other factors (38). LP is triggered by specific microbial polysaccharides. AP precedes CP and LP and is slowly self-activated by hydrolysis (38).

The three pathways mentioned above initiate complement activation, which converges on the common core component C3. C3 is cleaved into C3a and C3b by C3(H_2_O)Bb, which initiates signal amplification (39). CFD plays a promoting role in the generation of C3(H_2_O)Bb. In addition, initial pathways activation results in C5 convertase formation, which causes C5 to be cleaved into C5a and C5b. Moreover, the cleavage of C5 results in a labile conformation of C5b, which then binds successively to C6, C7, C8, and multiple copies of C9 to form the TCC, which is also called as the membrane attack complex (MAC) (37). The MAC can directly create transmembrane pores on cells and bacteria, leading to target swelling and eventual lysis (37).

Since complement cascade activation is a rapidly amplifying proinflammatory response, a variety of proteins that inhibit this reaction tightly regulate complement system activation in multiple ways, such as by modulating convertase activity and controlling the formation of the MAC. Complement factor I and complement factor H can limit the activity of C3 convertase and bind to C3b, preventing novel convertase formation (40).

### 3.2 The alternative pathway

AP activation starts with the slow spontaneous hydrolysis of C3 to form C3(H2O). MASP-3 is the exclusive activator of pro-CFD in resting human blood (41), promoting the proteolytic processing of pro-CFD to CFD (42). CFD cleaves C3(H2O) combined with CFB to the AP liquid phase C3 convertase C3(H_2_O)Bb. Through catalysis, C3 is cleaved into C3a and C3b. AP generates the majority of C3b (38). C3b later binds to a surface and recruits CFB to generate the C3bB complex. Subsequently, CFD, which binds to C3bB, becomes transiently active and cleaves CFB into Ba and Bb fragments, generating the AP cell surface C3 convertase C3bBb, which further promotes the generation of C3b. C3b binds to pathogens and acts as an opsonin to enhance phagocytosis. Additionally, when C3bBb accumulates and its local concentration reaches a certain level, C3b may bind to C3bBb, resulting in the generation of the C5 convertase C3bBb3b (43), which catalyzes downstream cascade reactions, resulting in the formation of MAC and lysis of the pathogen. Moreover, properdin combines with C3bBb to form C3bBbproperdin. This process stabilizes C3bBb, increasing its half-life by 5- to 10-fold (44).

### 3.3 The role of CFD in the amplification loop

In a variety of diseases, complement system activation leads to inflammatory damage, such as PNH and aHUS. Researchers have reported that it regulates the onset and development of ischemia/reperfusion (I/R) injury in recent years. Complement activation occurs early in acute myocardial infarction (AMI) (45). The elevation of C3d and sC5b9 occurs prior to the classical markers of myocardial necrosis in AMI patients (45).

AP has been regarded as a dual system (46). It both takes on a recognition function comparable to that of CP and LP and carries out an amplification function in the complement system. Since CP and LP activation generates C4bC2a, which forms C3b, triggering signal propagation via the amplification loop, AP amplification is a common feature of any kind of initial complement activation (46). AP amplification is responsible for approximately 80% of CP-induced complement terminal activation (47).

**Figure 3:**
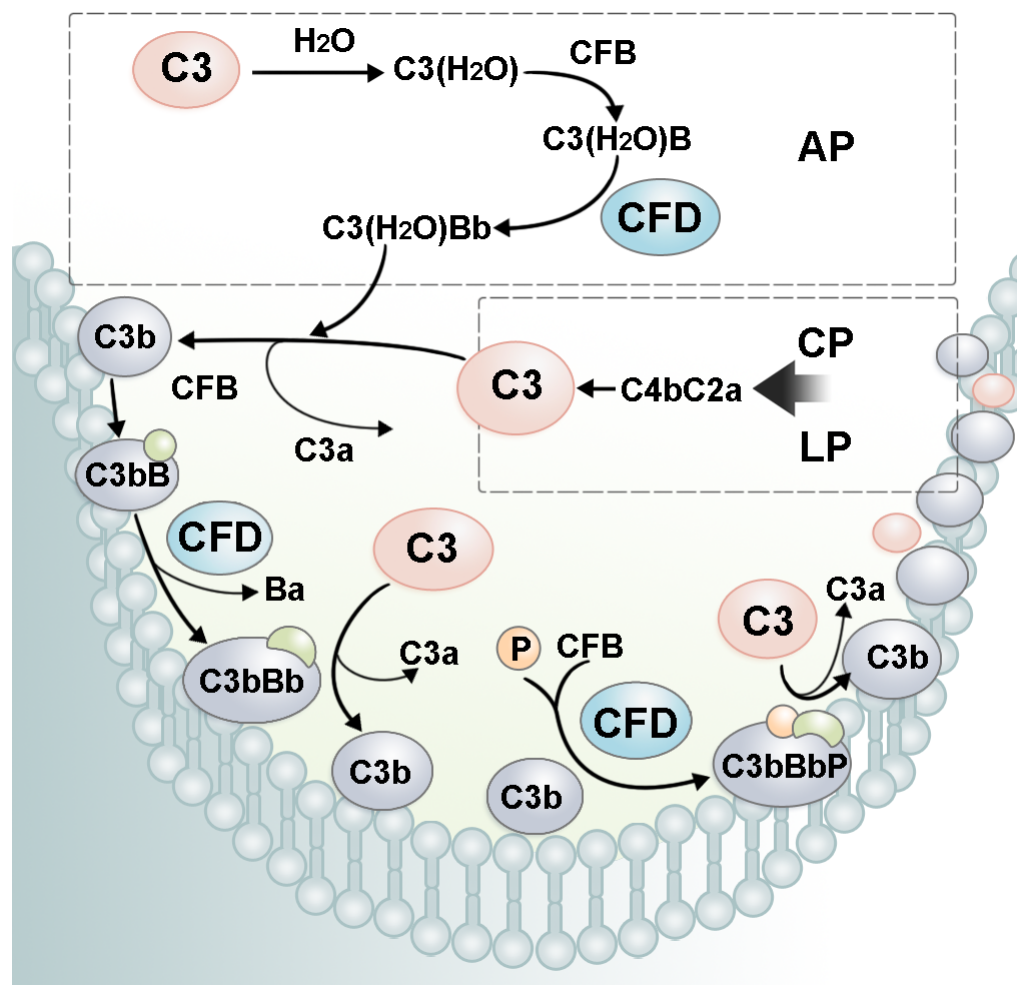
The role of CFD in the complement amplification loop. In AP, CFD cleaves C3(H_2_O)B into the alternative pathway fluid phase C3 convertase, C3(H_2_O)Bb. C3(H_2_O)Bb cleaves C3 into C3a and C3b. C3b recruits CFB on the cell surface. After cleavage by CFD, alternative pathway cell-surface C3 convertase, C3bBb, is formed. C3bBb cleaves C3 and forms more C3b, leading to a rapid amplification of complement cascade activity. P, properdin.

CFD is a pivotal rate-limiting enzyme in AP (**Figure 3**). The concentration of CFD is closely related to the activity and output of the AP and downstream amplification loop (7). The cleavage of CFB into Ba and Bb fragments by CFD is regarded as the rate-limiting stage for the generation of the C3 convertase C3(H_2_O)Bb. An increase in the CFD concentration enhances complement cascade activity. A lack of CFD has been shown to lead to inactive AP (22). More importantly, complement activation initiated by CP and LP can be amplified through the AP. C3b deposited on the cell surface by CP can serve as a site for AP C3 convertase formation (46). Under the catalysis of CFD, more C3bBb is produced and cleaves C3 to form more C3b deposited on the cell surface. Therefore, CFD amplifies the output of all three pathways through a positive feedback mechanism (48).

## 4 The role of CFD in cardiovascular diseases

**Figure 4:**
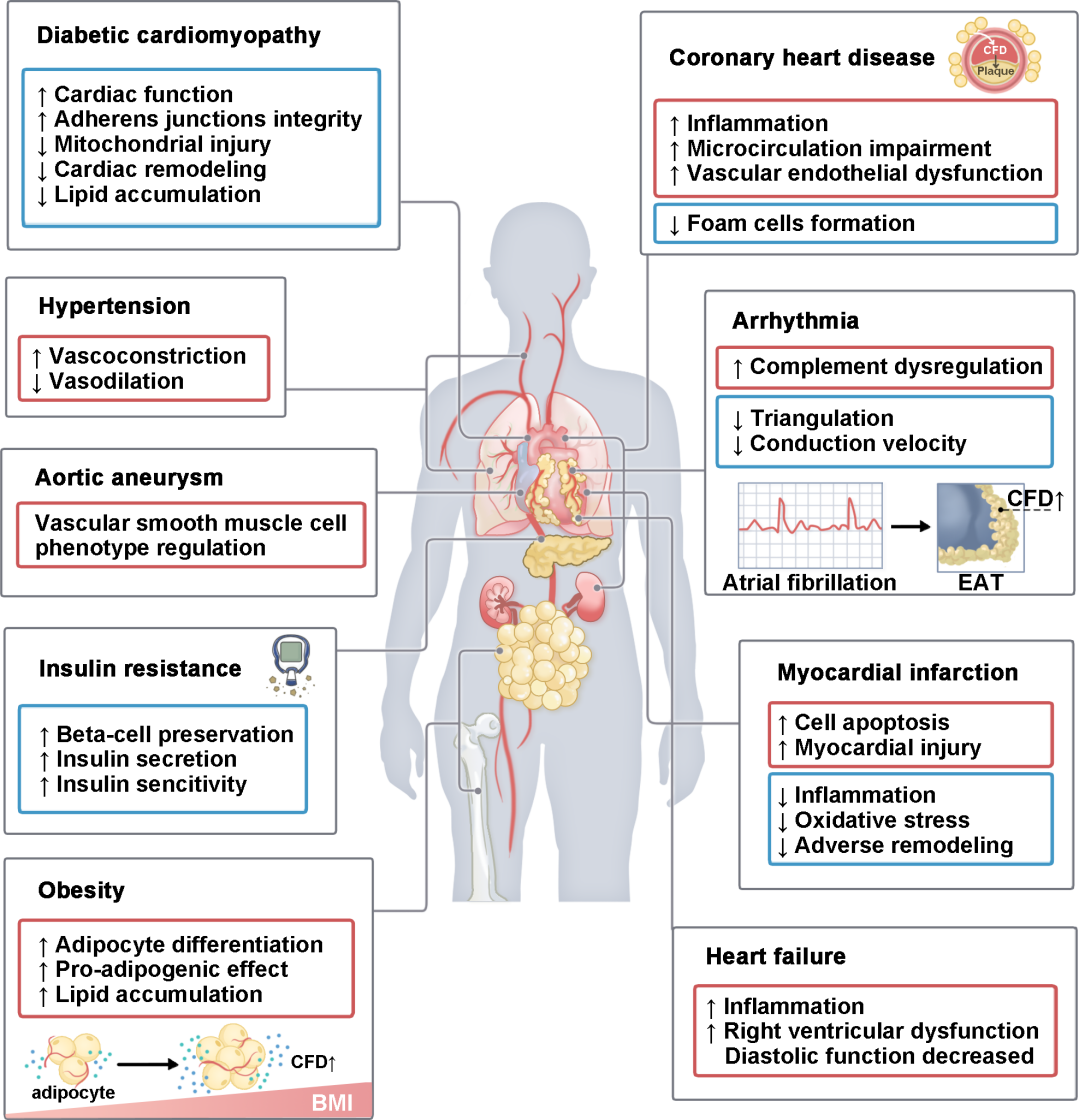
The role of CFD in various CVMDs. CFD plays a role in various CVMDs. It mainly includes diabetic cardiomyopathy, hypertension, aortic aneurysm, insulin resistance, obesity, coronary heart disease, arrhythmia, I/R injury and heart failure. This process involves multiple organs, including the heart, blood vessels, lungs, pancreas, kidneys, bones, and adipose tissue. In some diseases, CFD has both pathogenic and protective effects. BMI, body mass index; EAT, epicardial adipose tissue.

The findings summarized above emphasize the role of CFD in the complement system. We will briefly summarize the evidence that suggests possible roles of CFD (**Figure 4**) in each disease in the following text.

### 4.1 Hypertension

Many studies have verified the vital role of immune cytokines in hypertension and end-organ damage. Triggered by lifestyle factors, infections and autoimmunity, both chronic initiation and adaptive immune overactivation may result in high blood pressure and tissue damage (49). According to the Maastricht Study (4), participants with higher CFD are characterized by higher blood pressure. As an endogenous vascular elastase, CFD mediates the onset and development of pulmonary hypertension (PAH) (50). Moreover, CFD contributes to an obesity-related decrease in endothelium-dependent vasodilation and endothelin-1-dependent vasoconstriction (51).

As an autoimmune disease, systemic sclerosis (SSc) can lead to varying degrees of involvement in connective tissues, which can cause PAH in patients. The progression of PAH is related to elevated serine elastase activity (52). In patients with systemic sclerosis, CFD is increased in serum and is positively correlated with right ventricular systolic blood pressure and PAH. Patients with elevated CFD levels are at higher risk for PAH-related cardiac dysfunction (53). Importantly, high CFD levels are more specific to PAH than to brain natriuretic peptide levels. Mechanistically, progressive PAH is often associated with abnormalities in the structure and function of vascular and endothelial cells. In SSc, Friend leukemia virus integration 1 deficiency upregulates CFD in human dermal microvascular endothelial cells (19).

Innate immune response activation occurs when the embryo attaches to the uterus. Recent investigations have demonstrated that complement system dysregulation mediates the onset of preeclampsia, which is mainly characterized by gestational hypertension. CFD concentrations are reportedly increased in patients with preeclampsia during late gestation (54, 55). The concentration of complement terminal complexes in the placenta of preeclamptic women is higher than that in the placenta of normal women.

### 4.2 Coronary heart disease

CFD is a risk factor for coronary heart disease (CHD) in postmenopausal women (56). According to a cross-sectional study among 288 women, serum CFD levels are elevated in patients with polycystic ovary syndrome and are influenced by BMI, homeostatic model assessment for insulin resistance and high-sensitivity C-reactive protein (57). Another study showed that CFD can serve as an independent predictor of carotid intima-media thickness (CIMT) (57). Furthermore, CFD may be correlated with the instability of atherosclerotic plaques in patients with CHD (58). A study conducted among 370 patients undergoing diagnostic coronary angiography demonstrated that serum CFD levels have the potential to predict future incidences of AMI and all-cause death in patients with coronary artery diseases (58). CFD is highly expressed in coronary atherosclerotic plaques and adventitial adipocyte tissue (58). This reflects the potential role CFD plays in the development of atherosclerosis.

CFD plays a pathogenic role in CHD through multiple pathways. First, EAT can release proinflammatory factors directly into the coronary lumen because it is close to myocardiocytes. Previous studies have demonstrated that circulating CFD is exposed to the vascular endothelial layer and is correlated with systemic AP activation, which is correlated with vascular endothelial dysfunction (59). Moreover, pericoronary adipose tissue is bidirectionally linked to endothelial dysfunction (60). In response to angiotensin II stimulation, CFD is overly expressed in glomerular endothelial cells and activates local complement, resulting in the formation and deposition of the MAC complex on endothelial cells and promoting vascular endothelial injury (61). Then, complement induces endothelial cells to release inflammatory cytokines, which may subsequently promote vascular dysfunction (23). An increasing level of CFD is related to low-grade inflammation and endothelial dysfunction (6). Coronary angiography assessments using optical coherence tomography have shown that serum CFD levels can partially predict thin-cap fibroatheromas, which are inflammatory plaques that are prone to cause acute coronary syndrome (62). In addition, atherosclerosis is usually associated with abnormal lipid metabolism, such as increased intracellular cholesterol uptake and decreased excretion. CFD is expressed in the aortic endothelium (63). The expression of CFD, C1q alpha and beta, C2, and C3 is increased in atherosclerotic carotid plaques from acute coronary syndrome patients primed with C5a and tumor necrosis factor followed by cholesterol crystals (64).

Overall, the majority of studies show a promoting effect of CFD on atherosclerosis development. However, some studies have suggested a negative correlation between CFD and arterial injury. In 483 obese adult subjects, circulating CFD concentrations are reduced in individuals with CIMT and asymptomatic carotid atherosclerosis (65). Moreover, the overexpression of CFD inhibits the development of atherosclerosis in mice (66). Mechanistically, CFD downregulates the PPARγ/CD36 pathway, suppresses oxidized low-density lipoprotein-induced lipid uptake by macrophages, and prevents the formation of foam cells (66). Therefore, more in-depth investigations are still required to determine the specific mechanism of CFD in the onset and progression of CHD.

### 4.3 Ischemia/reperfusion injury

I/R injury can occur during myocardial infarction (MI) or cardiovascular surgery. AMI can lead to the production and secretion of CFD, and the serum CFD level of patients with AMI is upregulated at admission (17). At the 1-month follow-up, this difference in CFD levels between AMI patients and the control group disappeared (17). Among MI rats, the CFD expression levels in the EAT are markedly greater than those in subcutaneous adipose tissue. Inhibiting CFD activity can alleviate myocardial injury after MI (67). CFD derived from EAT induces cell apoptosis through excessive activation of poly ADP-ribose polymerase-1 in H9c2 cells (67).

However, studies have also suggested that CFD has a cardioprotective effect during MI injury by reducing inflammation, alleviating oxidative stress, and improving adverse remodeling. Open heart surgery may induce I/R injury. In 32 patients who received elective on-pump coronary artery bypass grafting with cardiopulmonary bypass, plasma CFD declined after surgery and decreased to the lowest level (approximately 30% below the baseline values) at 24 hours after surgery (68). There is an inverse correlation between CFD and IL-6 levels in samples collected at the start of cardiopulmonary bypass and 1 minute after removal of the aortic cross-clamp (68). In one study, CFD overexpression significantly increased the survival rate, preserved the left ventricular ejection fraction, and alleviated myocardial pathological damage in mice after MI surgery (17). CFD alleviates lipid oxidative stress after MI by decreasing cyclooxygenase-2 expression and increasing glutathione peroxidase 4 levels (17). Further observations noted that the CFD treatment of neonatal mouse ventricular myocytes altered the expression of proteins related to ferroptosis and iron metabolism (17). Mechanistically, CFD interacts with IRP2 to regulate iron homeostasis by upregulating the iron storage-related protein ferritin heavy chain and downregulating the transferrin receptor, thereby protecting cardiomyocytes against ferroptosis and reducing cardiac remodeling after MI (17).

### 4.4 Heart failure

Experimental and clinical evidence points to inflammatory mediators as a major cause of the development of heart failure (HF), as they can lead to maladaptive processes within the myocardium (39). Uncontrolled complement activation can cause widespread inflammation and tissue damage in HF. C3 and CFD are highly abundant in the right ventricle (69). In HF patients, the plasma concentration of CFD is elevated, especially in those with New York Heart Association functional class III and IV. The concentrations of CFD and terminal complement complexes are associated with the N-terminal pro-B-type natriuretic peptide and C-reactive protein concentrations (70). Increased CFD levels are also correlated with measures of inflammatory response and deteriorated diastolic function (70). In line with this, increased CFD is closely associated with left ventricular dysfunction (p < 0.001), right ventricular dysfunction, and a higher frequency of diastolic dysfunction in patients with systemic sclerosis (53). In conclusion, the concentration of CFD is correlated with the disease severity of patients with HF.

Recently, research has demonstrated that the C3-CFD-C3aR axis plays a crucial role in right ventricular failure. C3a-C3aR1 activated extracellular signal-regulated kinase and upregulated HF- and inflammation-related pathways in rat cardiomyocytes. In mouse models of right ventricular failure, both C3 knockout and CFD knockout attenuate ventricular dysfunction (69).

### 4.5 Arrhythmia

EAT accumulates and expands in obese and insulin-resistant states. In addition to functioning as an energy reserve system, epicardial fat determines the local inflammatory milieu of the heart via the release of multiple inflammatory and anti-inflammatory adipokines. The expansion of EAT can be proarrhythmic, resulting in hypoxia, inflammatory cytokine release, and the infiltration of fat into the myocardium. Epicardial fat increases the occurrence of atrial arrhythmias (71). Gene expression analysis revealed that the CFD gene was highly abundant in the EAT of patients with atrial fibrillation (AF) (72).

However, another study noted that the expression of CFD is downregulated in patients with AF-related cardiogenic embolism stroke (AF-CE) (73). Although CFD is associated with neutrophils, it is negatively correlated with naive B cells, naive CD4+ T cells, and resting NK cells. Bioinformatic analysis and experiments further support that CFD can potentially serve as a diagnostic blood biomarker for AF-CE (73). Thus, the decrease in CFD may imply complement dysregulation, which predicts the risk of AF-CE.

In direct coculture, white fat-like adipocytes (hAdip) increase triangulation, prolong action potential duration, and slow the conduction velocity of human-induced pluripotent stem cells to ventricular cardiomyocytes (74). The presence of MCP-1, IL-6, IL-8, and CFD, which are correlated with the immune response and inflammation, increased in the hAdip-conditioned medium. However, CFD and IL-8 can decrease triangulation and shorten conduction velocity through increasing action potential duration 30 alone (74).

### 4.6 Aortic aneurysm

Abdominal aortic aneurysm is correlated with vascular smooth muscle cell apoptosis and inflammation, and chronic inflammatory reactions activate the AP and may lead to the progression of atherosclerotic aortic aneurysm aortic wall remodeling. CFD levels are elevated in patients with thoracic aortic aneurysms and abdominal aortic aneurysms (75, 76), suggesting that CFD may promote vascular smooth muscle cell phenotype regulation in the onset of aneurysms.

## 5 The role of CFD in metabolic diseases

### 5.1 Obesity and dyslipidemia

The expansion of adipose tissue is often accompanied by the accumulation of immune cells. Emerging evidence shows that obesity is correlated with chronic LGI. There is a positive correlation between C3 and various indicators of body fat (77). Studies have shown that the concentrations of CFD are correlated with the likelihood of obesity (78). In overweight/obese women with a BMI≥25 kg/m^2^, circulating CFD levels are higher than those in subjects with a BMI<25 kg/m^2^ (57). An increasing number of studies have shown that BMI is positively correlated with CFD (79). Placental macrophages Hofbauer cells secrete CFD. More CFD was released from the placenta of obese mothers than from that of lean mothers (27). Circulating fetal CFD is positively correlated with maternal BMI and maternal insulin resistance (27).

There is still controversy about the type of adiposity associated with CFD. On the one hand, a cross-sectional study suggested that CFD and leptin concentrations are positively associated with BMI and visceral fat mass in patients with cardiometabolic disorders (79). In adults with growth hormone deficiency (AGHD), serum CFD is correlated with total body fat, visceral fat, and insulin resistance. After growth hormone treatment and appropriate lipid-lowering therapy, CFD levels significantly decrease in visceral adipose tissue (80). In contrast, a study has shown that CFD is related to subcutaneous and intrathoracic adipose volumes yet shows no connection with visceral adiposity (78).

In contrast, some studies have found the opposite effect on CFD. Plasma CFD levels are significantly downregulated in high-fat diet (HFD)-fed mice (81) but are slightly increased in isocaloric HFD-fed mice (82). Intriguingly, it has also been reported that CFD levels rise slightly within 48 hours after HFD feeding in mice but then decrease below baseline levels after 6 weeks after initiation of a HFD (83). Plasma proteome analysis has shown that preventative exercise can only partially reverse the decrease in plasma CFD caused by HFD overconsumption (82). Exercise inhibits the CFD-secreted phosphoprotein 1 signaling pathway in diet-induced obese male mice (84). Promoting the browning of white adipose tissue is considered a potential strategy for protecting against obesity. Transforming growth factor-beta, which is derived from macrophages, suppresses CFD mRNA and browning-related gene expression in adipocytes by activating Notch-1 signaling (81).

### 5.2 Hyperglycemia and insulin resistance

CFD plays an important role in promoting insulin secretion, regulating blood glucose levels and improving insulin sensitivity. In plasma, the level of CFD is decreased in patients with type 2 diabetes mellitus (T2DM) (85). Compared to T2DM patients, T2DM patients with β-cell failure exhibit decreased levels of CFD mRNA in visceral and subcutaneous adipose tissues (30). Animals with genetically absent CFD have glucose intolerance because of insulinopenia (30). In diabetic mice, CFD preserves beta cells by catalyzing C3a formation and downregulating dual specificity phosphatase 26 expression in β cells (78). Furthermore, CFD activates C3a-C3aR1 to promote insulin secretion (30). In addition, insulin promotes CFD secretion from adipocytes by stimulating phospholipase D activity (86).

### 5.3 Diabetic cardiomyopathy

Diabetic cardiomyopathy causes fatty acid and lipid accumulation in cardiomyocytes, resulting in lipotoxicity and triggering progressive structural and functional remodeling of the heart. In diabetic cardiomyopathy mice, CFD levels in heart tissue, plasma, white adipose tissue, and brown adipose tissue are significantly decreased (87). Overexpression of CFD relieves cardiac remodeling and improves cardiac function both in mice and rats with diabetic cardiomyopathy (87, 88).

CFD alleviates diabetes-related coronary microvascular complications (87). Specifically, CFD maintains the integrity of cardiac microvascular endothelial cell adherens junctions by downregulating tyrosine-protein kinase Csk and inhibiting VE-cadherin phosphorylation, thereby protecting the microvasculature against hyperpermeability (87). The abnormal processing of mitochondrial calcium and decreased level of free matrix calcium lead to a decrease in cardiomyocyte function. Recent research has revealed that CFD binds to interleukin-1 receptor-associated kinase 2 (Irak2) and inhibits its mitochondrial translocation, downregulating the interaction of Irak2 with prohibition-optic atrophy protein 1 on mitochondria in cardiomyocytes of mice treated with DMC. Inhibition of the Irak2 signaling pathway improves fatty acid oxidation, protects mitochondrial structure and function, and alleviates lipid accumulation (88).

## 6 Potential therapeutic strategies targeting CFD

The central role of CFD in AP activation and the critical role of CFD in complement cascade amplification make this protein an effective pharmaceutical target for complement-related diseases. As an inhibitor targeting the proximal part of AP, CFD inhibitor selectively block AP activation, impede C3 cleavage, and limit signal amplification of the CP and LP without any effect on the initiation or terminal function of these two complement pathways (8). Since the CP and terminal pathway mediate the bacterial lysis, CFD inhibition potentially reduces the risk of infections compared with C5 blockade or even C3 blockade (31). Previous studies have shown that terminal complement deficiencies patients have increased risk of infection with Neisseria species compared to early complement pathway component deficiencies (89). Additionally, C5a participate in the activation of leukocytes to phagocytose pathogens (90). Moreover, inhibiting CFD does not block C3b-mediated opsonization or C3a-mediated inflammation. Thus, CFD inhibitor modulate complement activation while keep “protective” complement activation.

Although CFD inhibitor have the advantage of oral administration, its short half-life requires 2–3 administrations per day (91). In addition, since that low levels of CFD are sufficient for AP activation in serum, complete inhibition of CFD may be vital for therapeutic efficacy (8). Compared to C5 inhibitors targeting downstream pathways, CFD inhibitors cannot fully block complement activation (91). C3 activation by other plasma protease, bypassing factor D, has been described in specific clinical circumstances (89). As a part of the plasma contact activation system, kallikrein cleaves C3 to C3b and C3a (89). These fundings suggest that a combined treatment with C5 inhibitors and CFD inhibitors may achieve better clinical response.

At present, a number of CFD inhibitors have been designed and quite a few of them have entered clinical trials. Benefited by its distinct mechanism, CFD inhibitors may achieve more profound clinical benefits in the future.

Danicopan (ALXN2040) is an oral CFD inhibitor that is mainly used as an add-on therapy in patients with PNH (NCT04469465). Danicopan improved hemoglobin concentrations with no serious adverse events reported in the study (92). Additionally, Danicopan is currently in a phase II clinical trial for patients with GA (NCT05019521). Furthermore, BCX9930 is another oral CFD inhibitor used in phase II clinical trials for the treatment of PNH (NCT04702568) and C3 glomerulopathy (NCT05162066).

There are few anti-CFD antibodies currently in clinical trials. Lanpazumab is a humanized monoclonal antibody targeting the antigen-binding fragment of CFD (93). In a cardiopulmonary bypass simulation, anti-CFD monoclonal antibodies inhibited the formation of inflammatory factors and the activation of neutrophils and platelets (36). Anti-CFD antibodies significantly inhibited the formation of alternative, lectin, and soluble TCCs in SARS-CoV-2-infected monkeys (94). In addition, CFD antibody treatment reduced the levels of proinflammatory cytokines, chemokines, and the plasma endothelial lesion marker plasminogen activator inhibitor-1 and improved coagulation abnormalities (94).

## 7 Conclusion and perspective

This review has shown the clear function of CFD in a range of CVMDs and identifies the role of CFD as a biomarker in these diseases. Current research shows that it has multiple effects on the cardiovascular system. Although CFD promotes endothelial dysfunction and atherosclerosis development and mediates ventricular diastolic dysfunction, it alleviates oxidative stress and improves adverse remodeling. Furthermore, regarding metabolic diseases, CFD promotes adipose tissue accumulation, controls blood glucose levels, improves insulin sensitivity and relieves diabetic cardiomyopathy. Thus, CFD may mediate interweaving immune responses and energy metabolism.

A large number of studies have confirmed the association between CFD and CVMDs. Many articles have pointed out the predictive value and biomarker role of CFD in CVMDs. However, the role of CFD in each CVMDS remains elusive. More research is required to clarify the effect of CFD on CVMDs and to confirm the specific mechanism by which CFD regulates CVMDs. Understanding the specific mechanism of CFD in the onset and development of diseases is an important knowledge base for discoveries about the diagnostic value and clinical treatment of CFD in CVMDS. Furthermore, researchers may adopt different methods to assess the safety, toxicology profile, and therapeutic effect of CFD-targeting medicine on CVMDS in animal models and human patients.

Overall, CFD is a prospective biomarker and therapeutic target for CVMDs.
